# Tip treatment for subnanoscale atomic force microscopy in liquid by atomic layer deposition Al_2_O_3_ coating

**DOI:** 10.1093/jmicro/dfaf014

**Published:** 2025-02-28

**Authors:** Ryohei Kojima, Ayhan Yurtsever, Keisuke Miyazawa, Lucas J Andrew, Mark J MacLachlan, Takeshi Fukuma

**Affiliations:** Division of Nano Life Science, Kanazawa University, Kakuma-machi, Kanazawa 920-1192, Japan; Nano Life Science Institute (WPI-NanoLSI), Kanazawa University, Kakuma-machi, Kanazawa 920-1192, Japan; Nano Life Science Institute (WPI-NanoLSI), Kanazawa University, Kakuma-machi, Kanazawa 920-1192, Japan; Faculty of Frontier Engineering, Kanazawa University, Kakuma-machi, Kanazawa 920-1192, Japan; Department of Chemistry, University of British Columbia, 2036 Main Mall, Vancouver, British Columbia V6T 1Z1, Canada; Nano Life Science Institute (WPI-NanoLSI), Kanazawa University, Kakuma-machi, Kanazawa 920-1192, Japan; Department of Chemistry, University of British Columbia, 2036 Main Mall, Vancouver, British Columbia V6T 1Z1, Canada; Division of Nano Life Science, Kanazawa University, Kakuma-machi, Kanazawa 920-1192, Japan; Nano Life Science Institute (WPI-NanoLSI), Kanazawa University, Kakuma-machi, Kanazawa 920-1192, Japan; Faculty of Frontier Engineering, Kanazawa University, Kakuma-machi, Kanazawa 920-1192, Japan

**Keywords:** atomic force microscopy, atomic layer deposition, atomic-resolution imaging, chitin nanocrystals, tip treatment, mica

## Abstract

Atomic force microscopy (AFM) allows direct imaging of atomic- or molecular-scale surface structures in liquid. However, such subnanoscale measurements are often sensitive to the AFM tip properties. To overcome this problem, 30 nm Si-sputter coating was proposed, and its effectiveness in improving stability and reproducibility has been demonstrated in atomic-scale imaging of various materials. However, this method involves tip blunting, enhancing the tip-induced dilation effect. As an alternative method, here we investigate atomic layer deposition (ALD) Al_2_O_3_-coating, where the film thickness is atomically well-controlled. Our transmission electron microscopy, contact angle and force curve measurements consistently suggest that as-purchased tips are covered with organic contaminants, and the initial 20 cycles gradually remove them, reducing the tip radius (*R*_t_) and hydrophobicity. Further deposition increases *R*_t_ and hydrophilicity and forms an intact Al_2_O_3_ film over 50 cycles. We compared 50-cycle ALD-coated tips with 30 nm Si-sputter-coated tips in imaging mica and chitin nanocrystals (NCs). On mica, ALD coating gives slightly less stability and reproducibility in hydration force measurements than the Si sputter coating, yet they are sufficient in atomic-scale imaging. In imaging chitin NCs, ALD-coated tips give a less tip-induced dilation effect while maintaining molecular-scale imaging capability. We also found that 10-cycle-ALD coated tips covered with carbon give a better resolution and reproducibility in observing subnanoscale features at chitin NC surfaces. This result and our experience empirically suggest carbon-coated tips’ effectiveness in observing carbon-based materials.

## Introduction

Recent advancements in atomic force microscopy (AFM) [1] enabled true atomic- and molecular-scale imaging of surface structures in liquids not only by contact-mode AFM [2,3] but also by dynamic-mode AFM such as frequency modulation AFM (FM-AFM) [4,5], amplitude modulation AFM (AM-AFM) [6,7], phase modulation AFM (PM-AFM) [8] and bimodal AFM [9,10]. These methods have been widely used for subnanoscale studies on various minerals [4,11–13], organic molecules [14–17] and biological systems [18–21]. Furthermore, combined with three-dimensional (3D) tip scanning methods, these methods made it possible to visualize 3D distributions of hydration structures [22–26], flexible molecular chains [27] and molecular adsorption structures [28,29]. However, such subnanoscale AFM images are sensitive to the tip apex structure and properties. Thus, using as-purchased tips without any treatment can lead to poor reliability, reproducibility and efficiency.

To solve this problem, various tip treatment methods were developed. For vacuum AFM, it has been common to clean the tip apex by irradiating Ar ion beam in the vacuum chamber. Furthermore, in low-temperature AFM, attaching a carbon monoxide molecule onto the tip apex has become a common practice [30], achieving an atomically well-defined probe. In contrast, for in-liquid AFM, tip treatment methods were mostly tested with force measurements [31,32] or nanoscale imaging [33] and atomic-scale studies are very limited [34]. As-purchased tips are typically covered with organic contaminants, making it difficult to perform atomic-scale AFM imaging. In practice, the tip surface is often crashed with the sample surface during the tip coarse approach or AFM imaging, exposing the tip material at the apex. However, the controllability and reproducibility of such an accidental removal of surface contaminants are not very high. Therefore, it has been strongly demanded to establish a well-controlled tip treatment method for atomic-scale in-liquid AFM.

So far, we have compared Ar sputter cleaning, Ar ion beam irradiation, UV/ozone cleaning and Si sputter coating [34]. While all the methods can successfully remove the tip surface contaminants, we found that the Si coating gives the best performance in atomic-resolution imaging and force curve measurements on mica in phosphate-buffered saline (PBS) solution. In addition, based on the results obtained by X-ray photoelectron spectroscopy and contact angle (CA) measurements, we proposed a hypothesis that the excellent performance of the Si sputter coating comes from the wet oxidation by the tip immersion into water and the resultant formation of stable hydration structures at the tip apex. Since then, we have demonstrated the effectiveness of this method by applying it to studies on inorganic [13], organic [28] and biological [35,36] samples.

However, this method has a major drawback: tip apex blunting. For example, we typically coat the tip with a 30 nm Si film [34], making the tip radius (*R*_t_) much blunter (∼30 nm) than that of as-purchased tips (*R*_t_ < 10 nm). While blunting is not an issue in imaging an atomically flat surface, it can be a serious problem in measuring the surface structures having a nanoscale corrugation. For example, cellulose or chitin nanocrystals (NCs) having a fibrillar shape with a diameter of several nanometers are imaged with a much larger width than actual due to the tip-induced dilation effect [35,36]: an increase in the apparent width of surface corrugations in an AFM image caused by the non-negligible tip geometry compared with the corrugation size [37,38]. Therefore, a tip treatment method without deteriorating the tip sharpness (*R*_t_ < 10 nm) is strongly demanded.

Recently, atomic layer deposition (ALD) has become a common technique for thin film formation [39]. ALD allows film formation with atomic-scale thickness control by repeating a cycle of precursor deposition and oxidation. In principle, this method should allow removing surface contaminants by oxidation and forming an intact hydrophilic film with a minimum thickness. However, the effectiveness of ALD coating on the tip treatment for atomic-scale in-liquid AFM has not been investigated.

In this study, we have investigated the effect of the tip treatment by ALD Al_2_O_3_ coating on atomic-/molecular-scale AFM experiments in liquid. First, we clarify the correlation between the number of ALD cycles and *R*_t_ by transmission electron microscopy (TEM) observations of tips coated with different thicknesses. Second, we clarify the minimum ALD cycles required for completely eliminating the surface contaminants and forming an intact Al_2_O_3_ film by CA measurements. Third, we investigate the performance of the ALD-coated tips in atomic-resolution imaging and force curve measurements on mica in PBS solution. Finally, we compare the Si sputtering and ALD coating in observing molecular-scale surface features and nanoscale outlines of chitin NCs in water.

## Methods

### Cantilever and tip treatments

In this study, we used Si cantilevers with backside Au coating (PPP-NCHAuD, Nanoworld). The cantilever’s nominal spring constant is 42 N/m. The typical resonance frequency and Q factor in liquid are 140 kHz and 8, respectively. The nominal *R*_t_ is <10 nm. 30 nm Si coating was performed with a DC sputter coater (KST-CSPS-KF1, K’s Tech). ALD Al_2_O_3_ coating was performed with SAL1000 (Suga Co., Ltd). Each ALD deposition cycle consists of precursor deposition and oxidation steps. In the former step, trimethyl aluminum (TMA) (Japan Advanced Chemicals) at 20°C was injected for 10 ms. In the latter step, Milli-Q water at 30°C was injected for 10 ms. The intervals between the steps were 15 s. During the whole process, N_2_ gas continuously flowed through the chamber at 50 sccm to eliminate the remaining precursor or water during the interval. Throughout the process, the sample stage and main ALD valve were kept at 300°C and 150°C, respectively.

All the ALD-coated tips used for the AFM experiments were not observed by TEM but directly transferred from the ALD chamber to the imaging solution through the air in 15–30 min. Although some organic contaminants may adsorb onto the tip apex during the transfer, they will likely desorb upon immersion into the water due to the hydrophilicity of an Al_2_O_3_ tip [34].

### TEM and CA measurements

TEM imaging was performed by JEM-2100Plus (JEOL) with a 200 kV acceleration voltage. A home-built sample holder was used for introducing a cantilever into the TEM chamber. We made the following efforts to minimize the carbon deposition during the TEM observations. We used a cold trap to keep the vacuum under 1.1–1.9 × 10^–5^ Pa. Adjustment of imaging conditions at high magnification was made by observing the edge of the upper part of the tip. Then, the imaging position was moved to the tip apex along the tip edge. As soon as it reached the tip apex, we blanked the electron beam (EB) and waited for 30 s to settle down the drift. Finally, we took the tip apex image with an EB exposure time of 0.17–0.2 s. From our experience, the carbon deposition under these conditions is small enough to estimate the thickness of the pre-existing contamination layer with nanoscale precision. CA measurements were performed by DM-301 (Kyowa) with a 2 µl milli-Q water droplet. The Si wafer used for the CA measurements was purchased from Nilaco (SI-500 443).

### Sample preparation

A 10 mM PBS solution was prepared by dissolving a PBS tablet (P4417-50TAB, Sigma-Aldrich) into 200 ml milli-Q water. A round disc of muscovite mica with a diameter of 12 mm (01877-MB, SPI Supplies) was cleaved by scotch tape. A 120 µl droplet of PBS solution was deposited onto the cleaved mica surface. FM-AFM force curve measurements and imaging were performed in the deposited solution.

The chitin NCs deposited on a mica surface were prepared as reported previously [35,40]. Briefly, to prepare aqueous dispersions of chitin NCs, an initial suspension of α-chitin (with a concentration of 3.5 wt. %) was first diluted to 0.002 wt. % using Milli-Q water. The resulting suspension was then sonicated with an ultrasonic homogenizer (MITSUI, 3 mm diameter tip, maximum power 500 W). To avoid excessive heating during this process, the chitin samples were placed in an ice bath and subjected to eight 5 min sonication cycles. A 120 µl drop of the diluted suspension (0.002 wt. %) was applied to a freshly cleaved mica substrate for AFM observation. Following a 20 min incubation period, the mica surface was thoroughly rinsed with Milli-Q water to further dilute the chitin concentration and eliminate any unattached chitin particles. A brief sonication step was performed prior to depositing the suspension on the mica substrate to prevent chitin NC aggregation. The prepared chitin NCs exhibited a relatively positive surface charge at pH 6–7, as confirmed by Zeta-potential measurements (35.1 ± 0.5 mV), and adhered strongly to the negatively charged muscovite-mica substrate through electrostatic forces. All sample preparations and experiments were carried out using Milli-Q deionized water with a resistivity of 18.2 MΩ.

### FM-AFM experiments

A home-built FM-AFM head with an ultra-low noise cantilever deflection sensor and a highly stable photothermal cantilever excitation system [41] was used. The system was controlled by a commercially available AFM controller (ARC2, Oxford Instruments) with customized software. A cantilever vibration was excited at its resonance frequency with a constant amplitude (*A*) using a phase-locked loop (PLL) circuit (OC4, SPECS). The PLL circuit was also used for detecting Δ*f*. The FM-AFM imaging was performed with a constant Δ*f* mode.

We made our best efforts to avoid the tip crash during the tip approach process as follows. In general, the Δ*f* signal decreases during the tip approach, and this decreasing speed becomes faster with the tip approach. In the first coarse approach step, the tip was quickly moved to the *z* position until the decreasing speed reached approximately −10 Hz/µm. In the second step, the tip was moved to the surface at ∼80 nm/s with the tip-sample feedback turned on. Under this condition, we can make a gentle tip approach without a tip crash.

## Results and discussion

### TEM observations

**Fig. 1.**
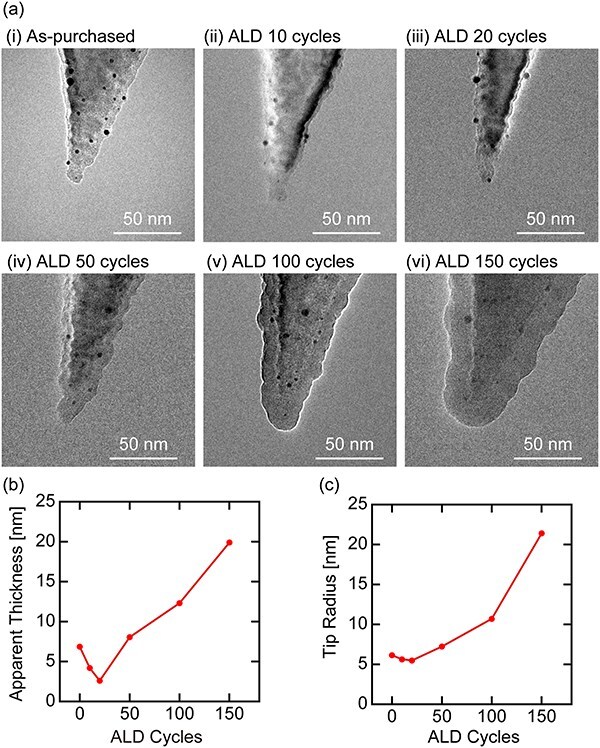
(a) TEM images of the tip apex with and without ALD treatments. ALD-cycle dependence of (b) Al_2_O_3_ film thickness and (c) *R*_t._

We prepared six cantilevers (PPP-NCHAuD, Nanoworld) and coated five of them with Al_2_O_3_ thin film by ALD with 10, 20, 50, 100 and 150 cycles. The tip apex of these cantilevers was observed with TEM as shown in Fig. 1a. Even without coating (Fig. 1a(i)), the tip apex appears to be covered with a layer showing a brighter contrast. This layer probably consists of a native oxide layer (i.e. SiO_2_) and hydrocarbon contaminants. Although the composition of the black dots observed on the tip surface is unknown, their dark contrast suggests that they are metal particles. The cantilever used in this experiment comes with a Au backside coating, which suggests that they may be Au particles.

For the initial 20 cycles (Fig. 1a(ii) and (iii)), the thickness of this bright layer and *R*_t_ are reduced, suggesting the elimination of the surface contaminants by oxidation. Above 50 cycles (Fig. 1a(iv)–(vi)), the thickness of the surface layer increases with ALD cycles. The deposition rate estimated by linear fitting to the data over 50 cycles was 0.119 nm/cycle, which agrees with the ideal rate (0.11–0.12 nm/cycle) reported for Al_2_O_3_ [42]. Thus, the stoichiometry of the observed layer should be Al_2_O_3_. At this stage, the black dots are either eliminated or embedded inside the Al_2_O_3_ layer, so that the tip apex should be free from their influence.

We estimated the apparent thickness (*t*_a_) of the bright layer at the tip side wall and *R*_t_ from individual TEM images (see Supplementary Figure S1 for more details) and plotted them against the ALD cycles as shown in Fig. 1b and 1c, respectively. These data confirm that both *t*_a_ and *R*_t_ decrease below 20 cycles and increase above that. The *t*_a_ data above 20 cycles shows an almost linear dependence on the ALD cycle, suggesting steady deposition of an Al_2_O_3_ film. Thus, the contamination removal process was probably changed to the Al_2_O_3_ deposition phase around 20 cycles. At this ALD cycle, *R*_t_ is expected to show the minimum value, ∼5 nm, as indicated in Fig. 1c, corresponding to the native *R*_t_ without contamination. This value is consistent with the manufacturer’s specification (i.e. *R*_t_ < 10 nm). As the native oxide layer under the carbon contaminants is expected to be 1–2 nm thick, the tip surface is mostly carbon under 20 cycles and Al_2_O_3_ above it. Only in a very limited condition of around 20 cycles, the tip surface may be SiO_2_. Overall, these results consistently suggest that 50 ALD cycles are sufficient to remove contaminants and form an intact Al_2_O_3_ film on the tip apex.

### CA measurements

**Fig. 2.**
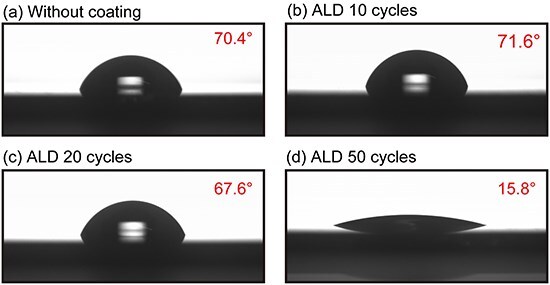
CA measurements of a water droplet deposited on a Si wafer with and without ALD Al_2_O_3_ coating. (a) Without coating. (b) 10 Cycles. (c) 20 Cycles. (d) 50 Cycles.

To gain insights into the hydrophilicity of the ALD-coated Si tip, we measured CAs of a water droplet deposited on a Si wafer with and without ALD Al_2_O_3_ coating, as shown in Fig. 2a–d. Without coating (Fig. 2a), the CA was relatively high (70.4°), suggesting the tip hydrophobicity due to organic contamination. Even after 10 or 20 cycles, the CA remains almost the same: 71.6° and 67.6°, respectively. As discussed earlier, the surface of the 20-cycle ALD-coated tip can be carbon, SiO_2_ or Al_2_O_3_, depending on the initial thickness of the carbon contamination layer. The observed high CA for this experiment suggests that the surface was still terminated with carbon even after 20 cycles. After 50 cycles, the CA dramatically decreased to show 15.8°, suggesting the coverage of the tip apex by hydrophilic Al_2_O_3_ film. This result is consistent with our TEM observations shown in Fig. 1.

Note that the hydrophilicity of an Al_2_O_3_ surface can vary depending on the surface termination. For an ALD-deposited Al_2_O_3_, its surface can be terminated differently by either finishing with the precursor deposition or oxidation [42]. In this experiment, we finished with an oxidation to terminate the surface with OH groups [43]. Therefore, the deposited Al_2_O_3_ film’s surface should be hydrophilic.

### Force curve measurements

**Fig. 3.**
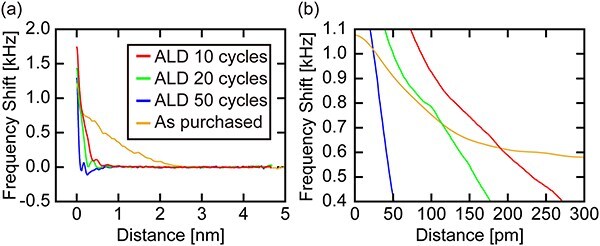
(a) Δ*f* versus distance curves averaged over 10 curves measured on mica in PBS solution by Al_2_O_3_-coated tips with different ALD cycles (*A* = 0.25 nm, tip velocity: 1 nm/s). All curves were obtained with the same Δ*f* setpoint of 1.954 kHz, yet the average curves end with different Δ*f* values. This is because a 50-point box filter was applied to each curve before averaging so that the initial and final 25 points were lost. The graph origin corresponds to the tip position, where Δ*f* reached the setpoint. (b) Magnified view of (a).

To investigate the influence of ALD Al_2_O_3_ coating on the frequency shift (Δ*f*) versus distance curve, we measured 10 Δ*f* curves on mica in PBS solution for each of the Al_2_O_3_-coated tips with different ALD cycles, and their average is plotted in Fig. 3a. The curve obtained with the as-purchased tip shows a relatively large long-range component over 2 nm range. This is probably due to the deformation of soft and swollen organic contaminants on the tip apex. Such influence of the long-range interaction was also observed in the energy dissipation curves simultaneously obtained with the Δ*f* curves as shown in Fig. S2. This long-range component was dramatically reduced by the 10 ALD cycles. This result suggests that the upper part of the contamination layer causing the long-range interaction was mostly removed after 10 cycles, although the surface is still covered with an organic layer as suggested by the TEM (Fig. 1a(ii)) and CA measurements (Fig. 2b). In addition, oscillatory profile corresponding to the hydration layer becomes visible over 10 cycles.

With increasing ALD cycles, Δ*f* slope near the surface becomes steeper. This trend is more clearly confirmed in Fig. 3b: a magnified view of Fig. 3a around Δ*f* of 0.8 kHz, a typical setpoint for atomic-resolution imaging of mica in liquid. In FM-AFM, the frequency noise (*δf*) multiplied by the Δ*f* slope at the setpoint determines the precision of the tip-sample distance control (*δz*). Thus, the higher Δ*f* slope gives the better *δz*. In this respect, the tip coated with 50 cycles should give the highest precision in the tip-sample distance control among the tested tips.

The increase in the Δ*f* slope was due to the thinning of the organic contamination layer by the oxidation step in the ALD cycle. As discussed earlier, the long-range interaction probably originates from the deformation of the soft and swollen organic contamination layer. Thus, its reduction makes the Δ*f* slope approach the value obtained with a SiO_2_ tip and eventually reach the one obtained with an Al_2_O_3_ tip. As SiO_2_ and Al_2_O_3_ are much more rigid than the swollen contaminants, their deformation or fluctuation is much smaller. Therefore, the Δ*f* slope increases with increasing the ALD cycles.

**Fig. 4.**
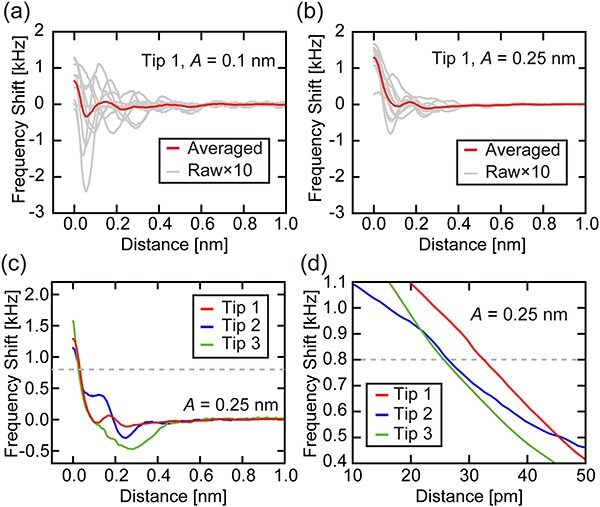
Δ*f* versus distance curves measured on mica in PBS solution using an Al_2_O_3_-coated tip with 50 ALD cycles. Raw and averaged curves measured with *A* of (a) 0.1 nm and (b) 0.25 nm. These measurements were performed with Tip 1. The same tip was also used to obtain the data shown in Fig. 3. (c) Δ*f* curves averaged over 10 curves obtained in each of Tips 1–3 performed with different tips. The average curve obtained with Tip 1 is identical to that shown in (b) and Fig. 3a. (d) Magnified view of (c) around Δ*f* of 0.8 kHz. All curves were obtained with the same Δ*f* setpoint of 1.954 kHz, yet they appear to end with different Δ*f* values. This is because a 50-point box filter was applied to each curve before averaging so that the initial and final 25 points were lost. The graph origin corresponds to the tip position, where Δ*f* reached the setpoint.

With 50-cycle ALD Al_2_O_3_-coated tips, we investigated the stability and reproducibility of the force curve measurements. First, we obtained 10 Δ*f* versus distance curves (Fig. 4a) with *A* = 0.1 nm, a typical value for hydration force measurements. Subsequently, we obtained another 10 curves with *A* = 0.25 nm (Fig. 4b), a typical value for atomic-resolution FM-AFM imaging in liquid.

With *A* = 0.1 nm (Fig. 4a), the individual curves (gray lines) show oscillatory profiles. However, their peak positions and heights show large variations. This may be partially due to the drift of the atomic-scale tip position. However, the observed variation is much larger than that reported for the 30 nm Si sputter coating [34]. In addition, a relatively large attractive peak was observed near the surface, which was previously reported as a common feature for an unstable tip [34]. Thus, the variation is likely to be also caused by tip instabilities. Despite the large variation, the average curve shows a clear repulsive peak at the vertical tip position (*z*) of 0.15 nm. This value agrees with the mean peak position statistically analyzed from the 10 curves: 0.16 ± 0.06 nm (mean ± standard deviation). As the estimated standard deviation is much smaller than the typical spacing between hydration peaks (0.2–0.4 nm), the peaks can be clearly seen in the average curve.

The variation in the force profiles is greatly suppressed by increasing *A* to 0.25 nm (Fig. 4b). For example, most curves show a peak at *z* = 0.18 nm. For both *A* values, averaged curves show similar oscillatory profiles with a hydration peak at *z* = 0.15–0.2 nm. Meanwhile, the peak becomes less evident by increasing *A*. This is reasonable as the sensitivity to the short-range interaction is reduced by increasing *A*. These results suggest that the ALD Al_2_O_3_-coated tip is applicable to hydration force measurements, yet its stability is not as high as Si-sputter-coated tips.

To investigate the reproducibility, we performed similar experiments three times with different ALD Al_2_O_3_-coated tips. For each experiment, we obtained 10 Δ*f* curves and their average is shown in Fig. 4c. For Tips 1 and 2, we see attractive and repulsive peaks at similar *z* positions. However, their magnitude is significantly different. For Tip 3, we only see a large and broad attractive peak and no hydration peak near the surface was observed. According to the previous study, where experiments and simulation of 3D-AFM imaging of hydration structures on calcite were compared in detail, the hydration force profile observed above the sharp repulsive branch is sensitive to the stability of the tip apex and hydration under it as well as lateral distribution of tip apex hydration [44]. Thus, the large variation of the hydration force profiles suggests the poor reproducibility of the ALD coating in terms of the atomistic tip apex structure or hydration.

Meanwhile, the Δ*f* slopes at Δ*f* = 0.8 kHz, a typical setpoint for atomic-resolution imaging of mica in PBS solution, are almost the same as shown in Fig. 4d: −24.1 ± 9.53, −18.3 ± 7.32 and −36.5 ± 28.9 kHz/nm for Tips 1–3, respectively. In the previous study, we estimated the minimum Δ*f* slope required for atomic-resolution FM-AFM imaging of mica under the same conditions to be ∼3 kHz/nm [34]. The Δ*f* slope values obtained by the three different tips consistently show a much higher slope. These results suggest 50-cycle ALD Al_2_O_3_-coated tips should be applicable to atomic-resolution mica imaging with sufficient reproducibility as we demonstrate in the following section.

### Atomic-resolution imaging of mica

**Fig. 5.**
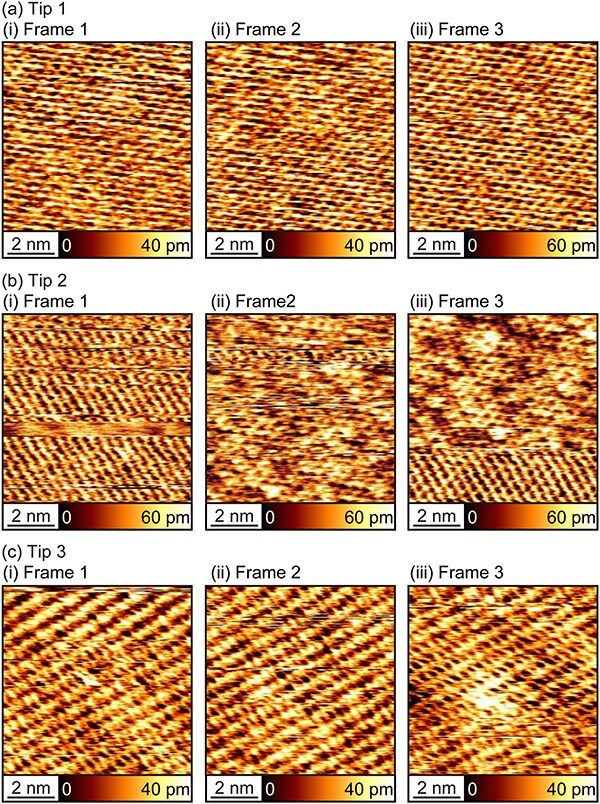
FM-AFM images of mica in PBS solution obtained with three different 50-cycle ALD Al_2_O_3_-coated tips (Δ*f* = 0.8 kHz. *A* = 0.25 nm. tip velocity: 223 nm/s.) For each tip, we obtained 10 frames just after the coarse tip approach, and the first three of them are shown here.

To investigate the applicability of 50-cycle ALD Al_2_O_3_-coated tips to atomic-resolution imaging in liquid, we performed FM-AFM imaging of mica in PBS solution with three different tips. In each experiment, we obtained the first 10 frames after a gentle tip approach to the surface with the same imaging conditions: Δ*f* = 0.8 kHz, *A* = 0.25 nm, and tip velocity (*v*_t_) of 223 nm/s. The first three frames out of the 10 frames are shown in Fig. 5.

For all three tips, the image showed atomic-scale contrasts from the first frame. For Tip 1, the image contrast showed almost no changes, suggesting high stability of the tip. For Tip 2, discontinuous changes of the image contrasts were observed for all three frames, suggesting the frequent changes in the tip apex structure. For Tip 3, although contrast changes were observed in the first and third frames, it was not as evident as observed with Tip 2 and it appears to be more continuous changes. Thus, the tip changes during the imaging were relatively minor. Comparing all the images, we found there are a few variations in the atomic-scale patterns such as dotted pattern (Fig. 5c(ii)), honeycomb-like pattern (Fig. 5b(iii)) and their intermediates (Fig. 5a(iii)). However, they are within the range of typical mica images, probably due to the subtle difference in tip apex structure and hydration.

Overall, these results suggest that the 50-cycle ALD Al_2_O_3_-coated tip can be used for atomic-resolution imaging in liquid with sufficient reproducibility. However, the stability of the atomic-scale contrasts during the imaging is not as stable as that obtained with 30 nm Si-coated tips.

### Molecular-scale imaging of chitin NCs

**Fig. 6.**
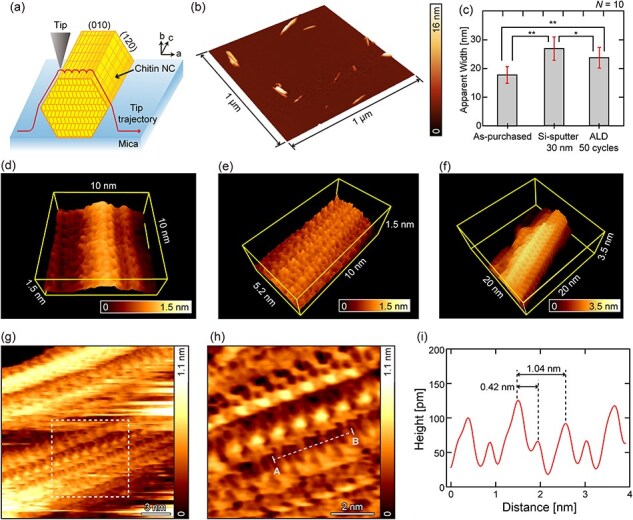
FM-AFM imaging of chitin NCs on mica in water. (a) Schematic illustration. (b) Large-scale image of the sample. (c) The *w*_a_ estimated from the images obtained with (i) as-purchased, (ii) Si-coated and (iii) 50-cycle ALD Al_2_O_3_-coated tips. The error bars indicate standard deviations. (d–h) FM-AFM images of chitin NCs obtained with (d) Si-coated, and (e) 50-cycle and (f–h) 10-cycle ALD Al_2_O_3_-coated tips. The white square in (g) indicates the scanned area for (h). (i) Height profile measured along line A-B shown in (h).

To examine the advantage of smaller *R*_t_, we compared 30 nm Si-sputter-coated and ALD Al_2_O_3_-coated tips in FM-AFM imaging of chitin NCs on mica in water, as shown in Fig. 6a. The chitin NCs used in this experiment have a diameter of several nanometers (∼7 nm on average) [35]. At the same time, their surface is molecularly flat, exhibiting subnanoscale corrugations. Thus, they are an ideal model sample for testing the imaging capability of nanoscale and subnanoscale surface structures with the same tip.

The large-scale image of the sample observed with a 50-cycle ALD Al_2_O_3_-coated tip is shown in Fig. 6b, where chitin NCs deposited on a mica surface are visualized. This image shows that the fibers are 100 nm—1 µm long. Meanwhile, due to the tip-induced dilation effect, their apparent width (*w*_a_) is typically larger than the actual width. From similar images obtained with as-purchased, Si-coated and 50-cycle ALD Al_2_O_3_-coated tips, we selected 10 single fibers with a height of 5.6 ± 1.2 nm and estimated their *w*_a_ as shown in Fig. 6c (see Supplementary Fig. S3 for more details). Among the three tips, the as-purchased tip gives the smallest *w*_a_, suggesting the tip blunting due to the Si or Al_2_O_3_ deposition. The ALD-coated tip provides smaller *w*_a_ than the Si-coated tip, demonstrating the advantage of the smaller *R*_tip_. However, in either case, *w*_a_ was larger than the expected diameter (∼5.6 nm) for the chitin NCs. Thus, the ALD-coating helps to suppress the tip-induced dilation effect yet cannot fully eliminate it.

A 30 nm Si sputter coated tip typically has *R*_t_ of ∼30 nm [34]. Thus, the observed average *w*_a_ of 26 nm seems too small. This can be explained by the existence of a small protrusion on the tip apex. An example of TEM images of a typical 30 nm Si sputter coated tip is shown in Fig. S4. Note that this is not the same tip used for this experiment. As you can see from these images, while overall *R*_t_ is ∼30 nm, there is a protrusion with a radius of 5.2 nm on the tip apex due to the relatively large surface roughness of the deposited Si film. This may serve as an effective tip for a surface corrugation with a height less than a few nm. Similarly, we consider that there was a protrusion with a ∼10 nm radius on the tip apex when we obtained the data for Fig. 6c. If that is the case, the protrusion is likely to be higher than the diameter of chitin NCs (5.6 ± 1.2 nm), so that it could effectively work as a tip, providing an apparent width of around 26 nm. However, as we cannot create such a protrusion in a controlled manner, the result is not reproducible. In addition, this does not work for a larger corrugation. In contrast, the ALD-coated tip shows a much smoother surface owing to the small roughness of the deposited Al_2_O_3_ film (Fig. 1a), providing a much better reproducibility.

To examine the tips’ molecular-scale imaging capability, we imaged the top surfaces of the chitin NCs with a 30 nm Si sputter coated tip (Fig. 6d) and a 50-cycle ALD-coated Al_2_O_3_ tip (Fig. 6e). These figures show that Si-coated and ALD-coated tips allow us to obtain clear molecular-resolution images. While we performed several experiments with each type of tip, we did not find differences between them. These results demonstrate that an ALD Al_2_O_3_-coated tip allows subnanoscale imaging of molecular surfaces while maintaining a relatively sharp tip apex.

In this study, we have mainly investigated the tips coated with an intact Al_2_O_3_ film by 50-cycle ALD in comparison with 30 nm Si sputter coated or as-purchased tips. Meanwhile, we unexpectedly found that a 10-cycle ALD-processed tip often provides a better resolution in the molecular-scale chitin NC imaging than the other types of tips as shown in Fig. 6f–h. Note that the tip surface is still covered with carbon even after a 10-cycle ALD, as shown by the TEM and CA measurements (Figs. 1 and 2). Figure 6g and its magnified image (Fig. 6h) show clear subnanoscale features of the chitin NC surface. The height profile taken along line A-B (Fig. 6i) shows a 1.04 nm periodicity along the fiber, consistent with the chitobiose unit size (∼1.04 nm). In contrast to the good regularity along the fiber, significant variations are observed in the perpendicular direction. This is consistent with our previous report, where such variations were attributed to the different rotation angles of the surface molecular chains [35].

The height profile in Fig. 6i also visualizes minor peaks separated by 0.42 nm from the major peaks. These results demonstrate the effectiveness of the 10-cycle coating in subnanoscale imaging of chitin NCs. Although similar features can be observed even with a Si-coated or 50-cycle ALD Al_2_O_3_-coated tip as we reported previously [35], the reproducibility and image quality are typically higher with 10-cycle ALD Al_2_O_3_-coated tips.

Although we do not understand the mechanism, we empirically have an impression that the carbon tips are unsuitable for atomic-resolution imaging of inorganic crystals [41] but often give a higher spatial resolution in imaging organic samples. Although subnanoscale imaging of organic materials by a carbon tip has not been reported yet, a large number of papers have been published on molecular-scale imaging of biological samples with an electron beam deposited (EBD) carbon tip [45,46]. Compared with the EBD method, 10-cycle ALD requires less expensive equipment and allows us to fabricate a sharper carbon tip with a higher controllability and reproducibility. While further systematic studies are required to clarify these advantages of the carbon tips and the underlying mechanism, this finding may potentially lead to an improvement in the imaging quality of organic samples.

## Concluding remarks

In this study, we have investigated the effect of the tip treatment by ALD Al_2_O_3_-coating on atomic- and molecular-scale AFM experiments in liquid. The TEM, CA, and force curve measurements consistently suggest that as-purchased tips are covered with organic contaminants, and the initial 20 cycles gradually remove them, reducing *R*_t_ and hydrophobicity. Further deposition increases *R*_t_ and hydrophilicity and forms an intact Al_2_O_3_ film over 50 cycles. We compared 50-cycle ALD coating with 30 nm Si-sputter coating in imaging mica and chitin NCs. On mica, in our previous report [34], Si-sputter coating allowed highly stable and reproducible force curve measurements and atomic-resolution imaging. Compared with that, ALD coating gives slightly less stability and reproducibility in hydration force measurements, yet they are sufficient in atomic-scale imaging. In imaging chitin NCs, ALD-coated tips give a less tip-induced dilation effect while maintaining molecular-scale imaging capability. We also found that 10-cycle ALD-coated tips covered with carbon give a better resolution and reproducibility in observing subnanoscale features at chitin NC surfaces. This result and our experience empirically suggest carbon-coated tips’ effectiveness in observing organic materials.

## Supplementary Material

dfaf014_Supplementary_Data

## References

[1] Binnig G , Quate C F and Gerber Ch (1986) Atomic force *microscope*. *Phys. Rev. Lett*. 56: 930–933. 10.1103/PhysRevLett.56.930 10033323

[2] Ohnesorge F and Binnig G (1993) True atomic-resolution by atomic force microscopy through repulsive and attractive forces. *Science* 260: 1451–1456. 17739801 10.1126/science.260.5113.1451

[3] Egger M, Ohnesorge F, Weisenhorn A L, Heyn S P, Drake B, Prater C B, Gould S A C, Hansma P K, and Gaub H E (1990) Wet lipid protein membranes imaged at submolecular resolution by atomic force microscopy. *J. Struct. Biol*. 103: 89–94. 10.1016/S0006-3495(90)82465-6 PMC1281069 2291944

[4] Fukuma T, Kobayashi K, Matsushige K, and Yamada H (2005) True atomic resolution in liquid by frequency-modulation atomic force microscopy. *Appl. Phys. Lett*. 87: 034101.

[5] Fukuma T, Kobayashi K, Matsushige K, and Yamada H (2005) True molecular resolution in liquid by frequency-modulation atomic force microscopy. *Appl. Phys. Lett*. 86: 193108.

[6] Voїtchovsky K, Kuna J J, Contera S A, Tosatti E, and Stellacci F (2010) Direct mapping of the solid-liquid adhesion energy with subnanometre resolution. *Nat. Nanotechnol*. 5: 401–405. 10.1038/nnano.2010.67 20418866

[7] Ricci M, Quinlan R A, and Voitchovsky K (2016) Sub-nanometre mapping of the aquaporin-water interface using multifrequency atomic force microscopy. *Soft Matter* 13: 187–195. 10.1039/c6sm00751a 27373564

[8] Fukuma T, Kilpatrick J I, and Jarvis S P (2006) Phase modulation atomic force microscope with true atomic resolution. *Rev. Sci. Instrum*. 77:043701. doi: 10.1063/1.2405361

[9] Martinez-Martin D, Herruzo E T, Dietz C, Gomez-Herrero J, and Garcia R (2011) Noninvasive protein structural flexibility mapping by bimodal dynamic force microscopy. *Phys. Rev. Lett*. 106: 198101. 10.1103/PhysRevLett.106.198101 21668203

[10] Martinez N F, Lozano J R, Herruzo E T, Garcia F, Richter C, Sulzbach T, and Garcia R (2008) Bimodal atomic force microscopy imaging of isolated antibodies in air and liquids. *Nanotechnology* 19: 384011. 10.1088/0957-4484/19/38/384011 21832570

[11] Rode S, Oyabu N, Kobayashi K, Yamada H, and Kuhnle A (2009) True atomic-resolution imaging of (1014) calcite in aqueous solution by frequency modulation atomic force microscopy. *Langmuir* 25: 2850–2853. 19437760 10.1021/la803448v

[12] Suzuki K, Oyabu N, Kobayashi K, Matsushige K, and Yamada H (2011) Atomic-resolution imaging of graphite–water interface by frequency modulation atomic force microscopy. *Appl. Phys. Express* 4: 125102.

[13] Miyata K, Tracey J, Miyazawa K, Haapasilta V, Spijker P, Kawagoe Y, Foster A S, Tsukamoto K, and Fukuma T (2017) Dissolution processes at step edges of calcite in water investigated by high-speed frequency modulation atomic force microscopy and simulation. *Nano Lett*. 17: 4083–4089. 10.1021/acs.nanolett.7b00757 28650174

[14] Hiasa T, Kimura K, and Onishi H (2012) Two-dimensional distribution of liquid hydrocarbons facing alkanethiol monolayers visualized by frequency modulation atomic force microscopy. *Colloids Surf., A* 396: 203–207.

[15] Hiasa T, Kimura K, and Onishi H (2012) Cross-sectional structure of liquid 1-decanol over graphite. *J. Phys. Chem. C* 116: 26475–26479.

[16] Asakawa H, Inada N, Hirata K, Matsui S, Igarashi T, Oku N, Yoshikawa N, and Fukuma T (2017) Self-assembled monolayers of sulfonate-terminated alkanethiols investigated by frequency modulation atomic force microscopy in liquid. *Nanotechnology* 28: 455603. 10.1088/1361-6528/aa8aa7 28876225

[17] Nishioka R, Hiasa T, Kimura K, and Onishi H (2013) Specific hydration on p-nitroaniline crystal studied by atomic force microscopy. *J. Phys. Chem. C* 117: 2939–2943.

[18] Hoogenboom B W, Hug H J, Pellmont Y, Martin S, Frederix P L T M, Fotiadis D, and Engel A (2006) Quantitative dynamic-mode scanning force microscopy in liquid. *Appl. Phys. Lett*. 88: 193109.

[19] Higgins M J, Polcik M, Fukuma T, Sader J E, Nakayama Y, and Jarvis S P (2006) Structured water layers adjacent to biological membranes. *Biophys. J*. 91: 2532–2542. 16798815 10.1529/biophysj.106.085688 PMC1562391

[20] Ido S, Kimura K, Oyabu N, Kobayashi K, Tsukada M, Matsushige K, and Yamada H (2013) Beyond the Helix Pitch: direct visualization of native DNA in aqueous solution. *ACS Nano* 7: 1817–1822. 23350676 10.1021/nn400071n

[21] Muller D J, Buldt G, and Engel A (1995) Force-induced conformational change of bacteriorhodopsin. *J Mol Biol* 249: 239–243. 10.1006/jmbi.1995.0292 7783190

[22] Fukuma T, Ueda Y, Yoshioka S, and Asakawa H (2010) Atomic-scale distribution of water molecules at the mica-water interface visualized by three-dimensional scanning force microscopy. *Phys. Rev. Lett*. 104: 016101. 10.1103/PhysRevLett.104.016101 20366372

[23] Kobayashi K, Oyabu N, Kimura K, Ido S, Suzuki K, Imai T, Tagami K, Tsukada M, and Yamada H (2013) Visualization of hydration layers on muscovite mica in aqueous solution by frequency-modulation atomic force microscopy. *J. Chem. Phys*. 138: 184704. 10.1063/1.4803742 23676061

[24] Marutschke C, Walters D, Walters D, Hermes I, Bechstein R, and Kuhnle A (2014) Three-dimensional hydration layer mapping on the (10.4) surface of calcite using amplitude modulation atomic force microscopy. *Nanotechnology* 25: 335703. 10.1088/0957-4484/25/33/335703 25074402

[25] Martin-Jimenez D, Chacon E, Tarazona P, and Garcia R (2016) Atomically resolved three-dimensional structures of electrolyte aqueous solutions near a solid surface. *Nat. Commun*. 7: 12164. 10.1038/ncomms12164 PMC4947176 27416784

[26] Kuchuk K and Sivan U (2018) Hydration structure of a single DNA molecule revealed by frequency-modulation atomic force microscopy. *Nano Lett*. 18: 2733–2737. 10.1021/acs.nanolett.8b00854 29564895

[27] Asakawa H, Yoshioka S, Nishimura K, and Fukuma T (2012) Spatial distribution of lipid headgroups and water molecules at membrane/water interfaces visualized by three-dimensional scanning force microscopy. *ACS Nano* 6: 9013–9020. 10.1021/nn303229j 23013290

[28] Ikarashi T, Nakayama K, Nakajima N, Miyata K, Miyazawa K, and Fukuma T (2022) Visualizing molecular-scale adsorption structures of anti-freezing surfactants on sapphire (0001) surfaces at different concentrations by 3D scanning force microscopy. *ACS Appl. Mater. Interfaces* 14: 44947–44957. 36126145 10.1021/acsami.2c10475

[29] Suzuki K, Kobayashi K, Oyabu N, Matsushige K, and Yamada H (2014) Molecular-scale investigations of structures and surface charge distribution of surfactant aggregates by three-dimensional force mapping. *J. Chem. Phys*. 140: 054704. 10.1063/1.4863346 24511965

[30] Gross L, Mohn F, Moll N, Liljeroth P, and Meyer G (2009) The chemical structure of a molecule resolved by atomic force microscopy. *Science* 325: 1110–1114. 19713523 10.1126/science.1176210

[31] Sirghi L, Kylian O, Gilliland D, Ceccone G, and Rossi F (2006) Cleaning and hydrophilization of atomic force microscopy silicon probes. *J. Phys. Chem. B* 110: 25975–25981. 10.1021/jp063327g 17181247

[32] Feng X, Kieviet B D, Song J, Schön P M, and Vancso G J (2014) Adhesion forces in AFM of redox responsive polymer grafts: Effects of tip hydrophilicity. *Appl. Surf. Sci*. 292: 107–110.

[33] Fujihira M, Okabe Y, Tani Y, Furugori M, and Akiba U (2000) A novel cleaning method of gold-coated atomic force microscope tips for their chemical modification. *Ultramicroscopy* 82: 181–191. 10741669 10.1016/s0304-3991(99)00144-8

[34] Akrami S M, Nakayachi H, Watanabe-Nakayama T, Asakawa H, and Fukuma T (2014) Significant improvements in stability and reproducibility of atomic-scale atomic force microscopy in liquid. *Nanotechnology* 25: 455701. 10.1088/0957-4484/25/45/455701 25327221

[35] Yurtsever A, Wang P X, Priante F, Morais Jaques Y, Miyata K, MacLachlan M J, Foster A S, and Fukuma T (2022) Probing the structural details of chitin nanocrystal-water interfaces by three-dimensional atomic force microscopy. *Small Methods* 6: 2200320. 10.1002/smtd.202200320 35686343

[36] Yurtsever A, Wang P X, Priante F, Morais Jaques Y, Miyazawa K, MacLachlan M J, Foster A S, and Fukuma T (2022) Molecular insights on the crystalline cellulose-water interfaces via three-dimensional atomic force microscopy. *Sci. Adv*. 8: eabq0160. 10.1126/sciadv.abq0160 PMC9565791 36240279

[37] Bonnet N, Dongmo S, Vautrot P, and Troyon M (1994) A mathematical morphology approach to image formation and image restoration in scanning tunnelling and atomic force microscopies. *Microsc. Microanal. Microstruct*. 5: 477–487.

[38] Flater E E, Zacharakis-Jutz G E, Dumba B G, White I A, and Clifford C A (2014) Towards easy and reliable AFM tip shape determination using blind tip reconstruction. *Ultramicroscopy* 146: 130–143. 24934394 10.1016/j.ultramic.2013.06.022

[39] George S M (2010) Atomic layer deposition: an overview. *Chem. Rev*. 110: 111–131. 10.1021/cr900056b 19947596

[40] Nguyen T D, Shopsowitz K E, and MacLachlan M J (2013) Mesoporous silica and organosilica films templated by nanocrystalline chitin. *Chem. Eur. J*. 19: 15148–15154. 24150881 10.1002/chem.201301929

[41] Fukuma T, Onishi K, Kobayashi N, Matsuki A, and Asakawa H (2012) Atomic-resolution imaging in liquid by frequency modulation atomic force microscopy using small cantilevers with megahertz-order resonance frequencies. *Nanotechnology* 23: 135706. 10.1088/0957-4484/23/13/135706 22421199

[42] Ott A W, Klaus J W, Johnson J M, and George S M (1997) Al303 thin film growth on Si (100) using binary reaction sequence chemistry. *Thin Solid Films* 292: 135–144.

[43] Lee K, Jur J S, Kim D H, and Parsons G N (2012) Mechanisms for hydrophilic/hydrophobic wetting transitions on cellulose cotton fibers coated using Al2O3 atomic layer deposition. *J. Vac. Sci. Technol. A Vac. Surf. Films* 30: 01A163. doi: 10.1116/1.3671942

[44] Miyazawa K, Tracey J, Reischl B, Spijker P, Foster A S, Rohl A L, and Fukuma T (2020) Tip dependence of three-dimensional scanning force microscopy images of calcite-water interfaces investigated by simulation and experiments. *Nanoscale* 12: 12856–12868. 32520063 10.1039/d0nr02043e

[45] Kodera N, Yamamoto D, Ishikawa R, and Ando T (2010) Video imaging of walking myosin V by high-speed atomic force microscopy. *Nature* 468: 72–76. 10.1038/nature09450 20935627

[46] Igarashi K, Uchihashi T, Koivula A, Wada M, Kimura S, Okamoto T, Penttila M, Ando T, and Samejima M (2011) Traffic jams reduce hydrolytic efficiency of cellulase on cellulose surface. *Science* 333: 1279–1282. 21885779 10.1126/science.1208386

