# Supplementary material for: Tip treatment for subnanoscale atomic force microscopy in liquid by atomic layer deposition Al2O3 coating
Source: Microscopy (Oxf). 2025 Feb 28;74(5):367–76. doi: 10.1093/jmicro/dfaf014 (PMC12527287; doi:10.1093/jmicro/dfaf014)
Supplement: dfaf014_Supplementary_Data [file dfaf014_supplementary_data.zip › suppl_data/Supplementary Data 20250218.docx]

**Supplementary Data**

**Tip treatment for subnanoscale atomic force microscopy in liquid by atomic layer deposition Al_2_O_3_ coating.**

Ryohei Kojima^1^, Ayhan Yurtsever^2^, Keisuke Miyazawa^2,3^, Lucas Andrew^4^, Mark J. MacLachlan^2,4^, and Takeshi Fukuma^1-3,^*

^1^Division of Nano Life Science, Kanazawa University, Kakuma-machi, Kanazawa 920-1192, Japan, ^2^Nano Life Science Institute (WPI-NanoLSI), Kanazawa University, Kakuma-machi, Kanazawa 920-1192, Japan, ^3^Faculty of Frontier Engineering, Kanazawa University, Kakuma-machi, Kanazawa 920-1192, Japan, ^4^Department of Chemistry, University of British Columbia 2036 Main Mall, Vancouver, V6T 1Z1 Canada

*Correspondence should be addressed to

Takeshi Fukuma, Nano Life Science Institute, Kanazawa University, Kakuma-machi, Kanazawa 920-1192, Japan.

Phone: +81-76-234-4847

E-mail: fukuma@staff.kanazawa-u.ac.jp

**Methods for estimating *R*_t_ and *t*_a_**

**_
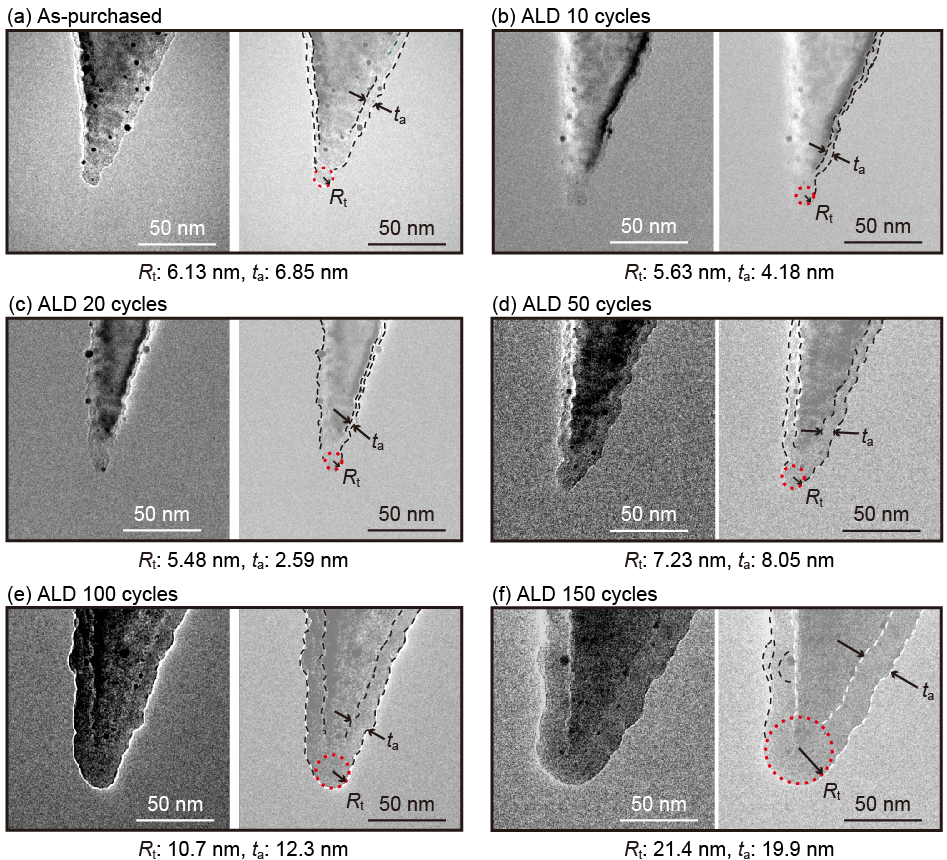
_**

**Fig. S1** Estimation of *R*_t_ and *t*_a_ from the TEM images of the tip apex with and without ALD treatments. The TEM images are the same as shown in Fig. 1(a) in the main text but with a different contrast and brightness.

The tip radius (*R*_t_) was estimated by fitting a circle to the tip apex outline as indicated by the red dotted lines in Fig. S1. Meanwhile, for estimating the apparent thickness (*t*_a_) of the overlaid layer on the tip sidewall, black dotted lines were drawn at the tip outline and the boundary between the Si and overlaid film either consisting of carbon contaminants or deposited Al_2_O_3_. For the latter, the dotted lines were indicated only for the locations, where contrast difference was evident. From these dotted lines, it is clear that the thickness is not constant but dependent on the position. Therefore, we defined *t*_a_ as the maximum of the apparent thickness as indicated by the arrows in Fig. S1.

**Energy dissipation curves**

**
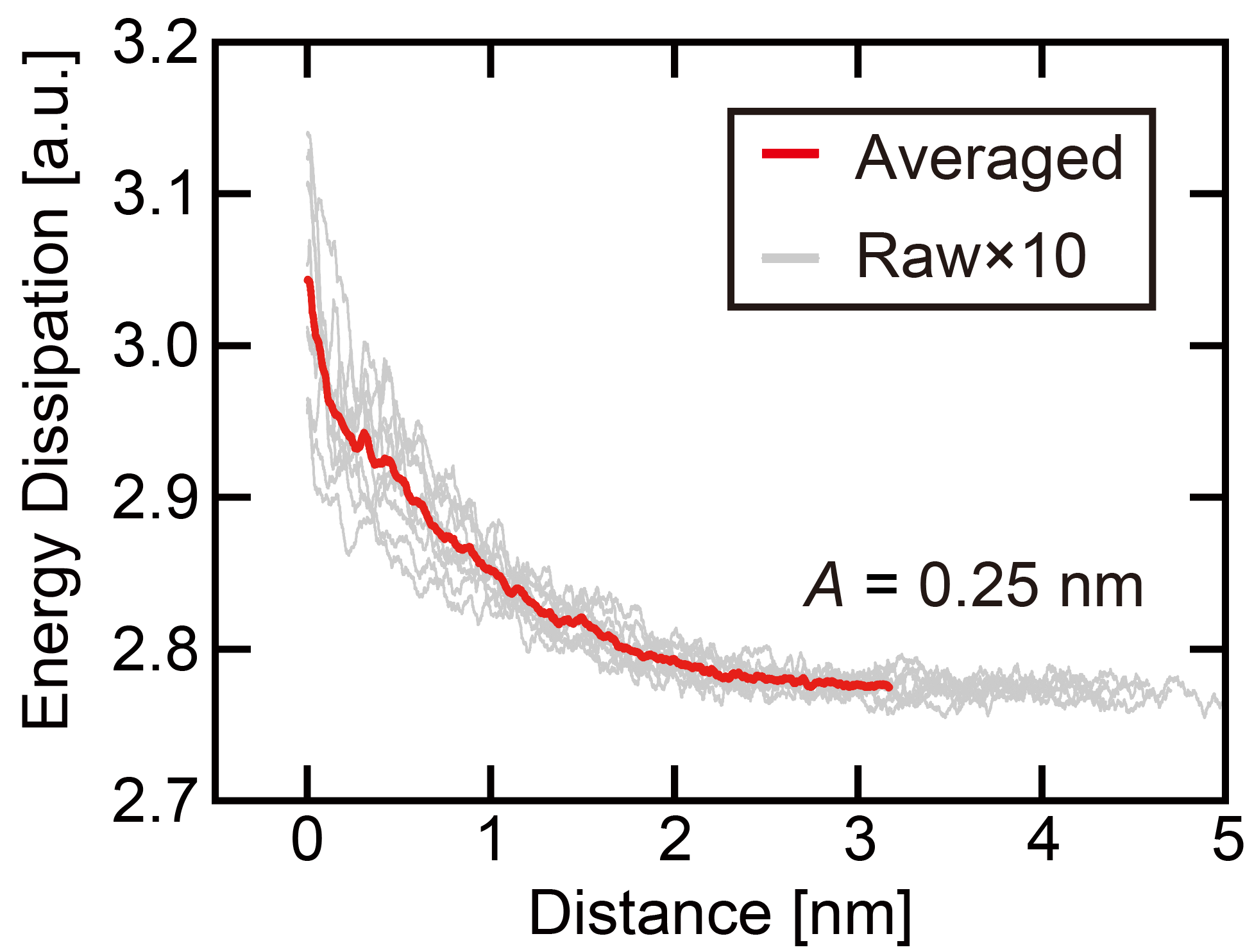
**

**Fig. S2** Energy dissipation curves obtained in PBS solution on mica by the 50-cycle ALD-coated Al_2_O_3_ tip. The gray curves are raw data while the red curve is their average. As one of the curves does not have data above 3.2 nm, the average curve starts from that distance. The average of the frequency shift curves simultaneously obtained with these curves is shown in Fig. 3a in the main text.

As the tip approaches the surface, energy dissipation gradually increases from ~3 nm away from the surface probably due to the deformation of soft and swollen organic contaminants on the tip surface. This result shows that the influence of such surface contamination can be detected not only by the frequency shift signal but also by the energy dissipation signal.

**Methods for estimating *w*_a_**


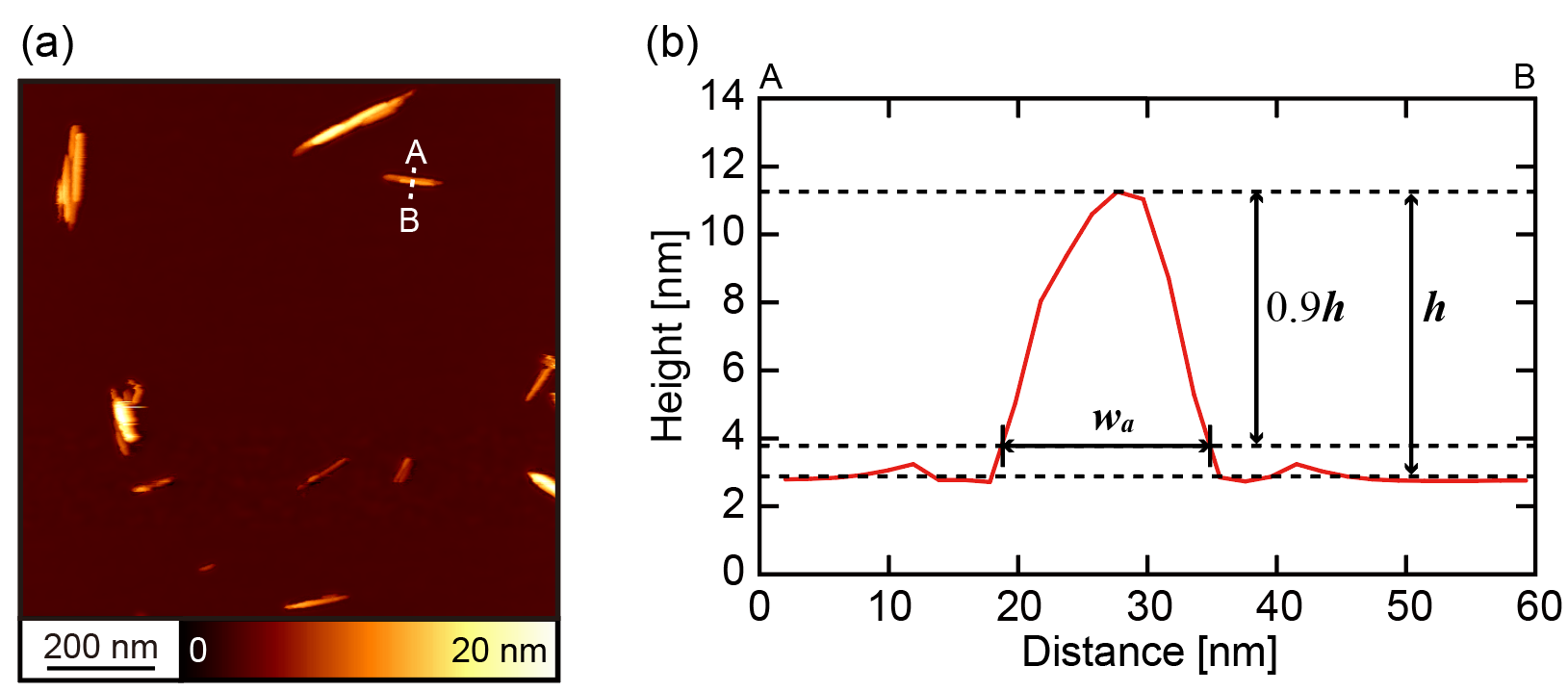


**Fig. S3** Method for estimating *w*_a_ from FM-AFM images of chitin NCs on mica in PBS. (a) FM-AFM Image of chitin NCs. This is a different view of the same image as shown in Fig. 6b. (b) Height profile measured along line A-B, where the definition of *w*_a_ is indicated.

For estimating the apparent width (*w*_a_) of the chitin NCs in the FM-AFM images, we took a cross-sectional height profile. An example of such an image and profile are shown in Fig. S3. As indicated in Fig. S3b, we defined *w*_a_ as the width at the 90% lower height than the peak.

**TEM image of Si sputter coated tip**


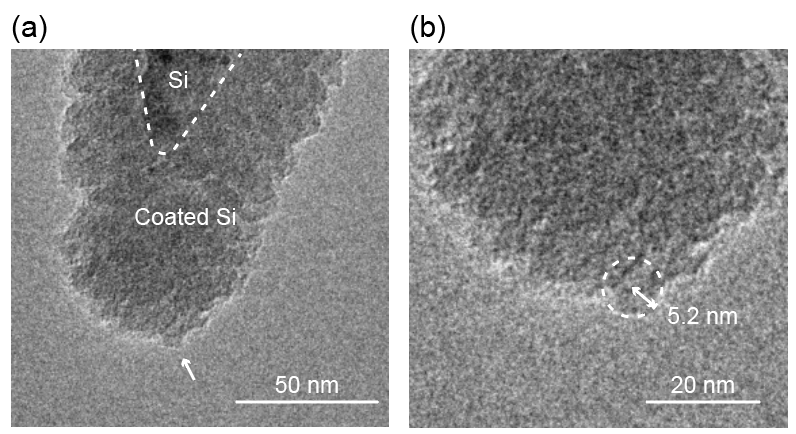


**Fig. S4** (a) TEM image of a typical 30 nm Si sputter coated tip. (b) Magnified view of (a). While the overall tip radius is around 30 nm, there are smaller protrusions as indicated by the arrow in (a) due to the rough surface of the deposited Si film. In this example, the topmost protrusion has a radius of 5.2 nm. This may effectively serve as a tip for surface corrugations with a depth of few nm. However, this is not the case for a larger corrugation. In addition, we cannot create such a protrusion in a controlled manner, so that the reproducibility is very poor.
